# Dynamics of DNA Methylation during Early Development of the Preimplantation Bovine Embryo

**DOI:** 10.1371/journal.pone.0066230

**Published:** 2013-06-14

**Authors:** Kyle B. Dobbs, Marlon Rodriguez, Mateus J. Sudano, M. Sofia Ortega, Peter J. Hansen

**Affiliations:** 1 Department of Animal Sciences, D.H. Barron Reproductive and Perinatal Biology Research Program, and Genetics Institute, University of Florida, Gainesville, Florida, United States of America; 2 Departamento de Reprodução Animal e Radiologia Veterinária, Faculdade de Medicina Veterinária e Zootecnia, Universidade Estadual Paulista, Campus de Botucatu, São Paulo, Brazil; Michigan State University, United States of America

## Abstract

There is species divergence in control of DNA methylation during preimplantation development. The exact pattern of methylation in the bovine embryo has not been established nor has its regulation by gender or maternal signals that regulate development such as colony stimulating factor 2 (CSF2). Using immunofluorescent labeling with anti-5-methylcytosine and embryos produced with X-chromosome sorted sperm, it was demonstrated that methylation decreased from the 2-cell stage to the 6–8 cell stage and then increased thereafter up to the blastocyst stage. In a second experiment, embryos of specific genders were produced by fertilization with X- or Y-sorted sperm. The developmental pattern was similar to the first experiment, but there was stage × gender interaction. Methylation was greater for females at the 8-cell stage but greater for males at the blastocyst stage. Treatment with CSF2 had no effect on labeling for DNA methylation in blastocysts. Methylation was lower for inner cell mass cells (i.e., cells that did not label with anti-CDX2) than for trophectoderm (CDX2-positive). The possible role for *DNMT3B* in developmental changes in methylation was evaluated by determining gene expression and degree of methylation. Steady-state mRNA for *DNMT3B* decreased from the 2-cell stage to a nadir for D 5 embryos >16 cells and then increased at the blastocyst stage. High resolution melting analysis was used to assess methylation of a CpG rich region in an intronic region of *DNMT3B*. Methylation percent decreased between the 6–8 cell and the blastocyst stage but there was no difference in methylation between ICM and TE. Results indicate that DNA methylation undergoes dynamic changes during the preimplantation period in a manner that is dependent upon gender and cell lineage. Developmental changes in expression of *DNMT3B* are indicative of a possible role in changes in methylation. Moreover, *DNMT3B* itself appears to be under epigenetic control by methylation.

## Introduction

Following fertilization, the embryo remains in a state of transcriptional quiescence that is maintained until a species-specific stage (8–16 cell stage in the cow, 2-cell stage in the mouse, 4-cell stage in the pig and 4–8 cell stage in the human) when transcription is resumed through a process referred to as embryonic genome activation [1]. In the mouse, activation of transcription is preceded by a decrease in global methylation of DNA as a result of active and passive demethylation that begins at the zygote stage and persists through the morula stage [2], [3]. Demethylation is followed by a wave of DNA methylation beginning at the blastocyst stage [4] that is mediated by *de novo* methyltransferases Dnmt3A and Dnmt3b [4], [5]. Overall levels of methylation in the blastocyst are greater for ICM than TE [4].

Epigenetic remodeling is important for embryonic genome activation because mouse embryos in which the chromatin remodeling gene, *Brg1*, was knocked out exhibited arrest at the 2-cell stage and decreased transcription [6]. Moreover, injection of siRNA for the H3K27me3 demethyltransferase *JMJD3* decreased competence of bovine embryos to develop to the blastocyst stage [7]. Nonetheless, developmental patterns of *de novo* methylation in early development are not conserved across mammalian species. The demethylation occurring during early cleavage-stages in the mouse also occurs in sheep [8] but not in the pig [9] or rabbit [10]. By the blastocyst stage, the ICM is more methylated than the TE in the sheep [8] and pig [9] while, in the rabbit, the ICM is less methylated than TE [10]. *De novo* methylation is not as clearly understood in other species. In the sheep, overall DNA methylation declines from the two-cell stage until the blastocyst stage and the ICM is more methylated than the TE [8]. The ICM is also more methylated than the TE in the pig blastocyst, but unlike the mouse and sheep, there is no apparent loss of DNA methylation from the two-cell to morula stages of development [9].

Results are unclear in the bovine embryo, with one report indicating widespread demethylation occurs through the 8-cell stage before methylation increases at the 16-cell stage of development [11] while another report indicates that demethylation persists through the morula stage [12]. The difference in methylation between the ICM and TE of the bovine blastocyst is also unclear, with one report indicating higher methylation in the ICM [11] and another indicating higher methylation in TE [12].

Developmental changes in the embryonic methylome are also likely to be modified by genetics of the embryo and the environment in which it resides. Genetic sex can have profound effects on development of the embryo as early as the blastocyst stage when, for example, total transcript abundance in cattle is higher in female blastocysts than male counterparts [13]. Expression of *de novo* methyltransferases is also differentially expressed between the two genders, with female blastocysts in the cow having lower expression of *DNMT3A* and *DNMT3B* compared to male blastocysts [14]. Signals derived from the mother can also affect embryonic development in a way that improves competence for survival after transfer into females, as has been demonstrated in the cow for CSF2 [15], insulin-like growth factor-1 [16] and hyaluronan [17]. The molecular basis for improvement in competence for long-term development is not known but could include remodeling of the epigenome. Indirect evidence for epigenetic programming during early development is the observation that treatment of embryos with CSF2 from Day 5–7 of pregnancy improves late embryonic and fetal survival much later in pregnancy (after Day 30–35 of gestation) [15].

In this study, we characterized dynamics of DNA methylation during early development in the cow and determined whether degree of methylation depends upon cell lineage (ICM vs TE), gender, and exposure to CSF2. In addition, developmental changes in expression and methylation of *DNMT3B* were assessed to determine whether developmental changes in DNA methylation could conceivably be due to differences in activity of this methylase.

## Experimental Procedures

### Embryo Production

*In vitro* production of bovine embryos was performed as previously described [18] unless otherwise noted. Cumulus oocyte complexes (COC) were obtained with permission by cutting the surface of slaughterhouse (Central Beef Packing Co. (Center Hill, FL, USA)-derived ovaries with a scalpel and vigorously rinsing the ovary through a bath of oocyte collection medium [tissue culture medium-199 with Earle’s salts without phenol red (Hyclone, Logan UT), 2% (v/v) bovine steer serum (Pel-Freez, Rogers, AR), 2 U/ml heparin, 100 U/ml penicillin-G, 0.1 mg/ml streptomycin and 1 mM glutamine]. Groups of 10 COC were matured in 50 µl droplets of oocyte maturation medium [tissue culture medium-199 with Earle’s salts (Invitrogen, Carlsbad, CA), 10% (v/v) bovine steer serum, 2 µg/ml estradiol 17-β, 20 µg/ml bovine follicle stimulating hormone (Bioniche Life Sciences, Belleville, Ontario, Canada), 22 µg/ml sodium pyruvate, 50 µg/ml gentamicin sulfate and 1 mM glutamine] covered with mineral oil. Maturation proceeded for 20 h at 38.5°C and in a humidified atmosphere of 5% (v/v) CO_2_. Up to 300 matured oocytes were fertilized with Percoll-purified sperm (1×10^6^) for 8 h in 1.7 ml of SOF-FERT [19]. Cumulus cells were denuded after fertilization for 4 min by vortexing in 600 µl HEPES-TALP [20] containing 10,000 U/ml hyaluronidase. Embryos were then cultured in 25 µl microdrops of SOF-BE1 [18] covered with mineral oil at 38.5°C in a humidified atmosphere of 5% (v/v) O_2_ and 5% (v/v) CO_2_ with the balance N_2_. Cleavage rate was assessed at d 3 post-insemination (pi) and embryos were cultured until d 7 pi.

### Immunofluorescent Labeling for 5-methylcytosine

All labeling steps were performed at room temperature unless otherwise stated and in a volume of 100 µl (either in microdrops covered with oil or in wells of a 96-well plate). Embryos were harvested from culture drops, washed in Dulbecco’s phosphate buffered saline (DPBS) containing 1% (w/v) polyvinylpyrrolidone (PVP) (Kodak, Rochester, NY, USA) and fixed in 4% (w/v) paraformaldehyde in DPBS-PVP for 15 min. The fixed embryos were washed three times in DPBS-PVP, permeabilized for 20 min in 0.25% Triton X-100 (Fisher Scientific, Waltham, MA, USA) diluted in DPBS, and washed three times in 0.1% (v/v) Tween 20 with wash buffer [DPBS containing 10 mg/ml fraction V BSA (Sigma-Aldrich, St. Louis, MO, USA)]. Embryos were then incubated for 1 h in 50 µg/ml RNase A (Qiagen, Valencia, CA, USA) diluted in DPBS-PVP at 37°C, washed three times in wash buffer and then incubated at 37°C for 30 min with 3 M HCl/0.1% (w/v) PVP. The pH was neutralized by incubation of embryos for 10 min with 100 mM Tris-HCl, pH 8.5 containing 1% (w/v) PVP followed by three washes with wash buffer.

Nonspecific binding sites were blocked by incubation with DPBS containing 5 mg/ml bovine serum albumin (BSA) for 1 h. Embryos were then transferred to a solution of 1 µg/ml anti-5-methylcytosine (affinity-purified mouse monoclonal antibody; (Calbiochem, Darmstadt, Germany) diluted in DPBS containing 0.05% (v/v) Tween 20 and 0.01% (w/v) BSA. As a negative control, anti-5-methylcytosine was replaced with an irrelevant mouse IgG1 antibody (Sigma-Aldrich, St. Louis, MO, USA). After 1 h, embryos were washed three times in wash buffer and transferred to 1 µg/ml fluorescein isothiocyanate (FITC) conjugated anti-mouse IgG (Abcam, Cambridge, MA, USA). After three washes, nuclei were labeled by incubation in 50 µg/ml propidium iodide (PI) diluted in DPBS-PVP for 15 min. Embryos were washed three times and mounted with coverslips on slides using ProLong Gold Anti-Fade mounting medium (Invitrogen, Carlsbad, CA, USA) and observed under a Zeiss Axioplan epifluorescence microscope (Zeiss, Göttingen, Germany). Images were acquired using a 40× objective and FITC, blue and rhodamine filters. The exposure times were constant for all embryos analyzed in an individual replicate.

### Immunofluorescent Labeling for 5-methylcytosine and CDX2

In one experiment, labeling with antibody to 5-methylcytosine was determined for ICM and TE using anti-CDX2 to label TE [21]. Embryos were labeled for anti-methylcytosine as described above, washed six times and transferred to a solution of antibody against CDX2 (affinity purified mouse monoclonal antibody against CDX-2, ready to use solution; BioGenex, San Ramon, CA, USA) for 1 h. Embryos were washed three times and transferred to 10 µg/ml Alexa Fluor 350 labeled goat anti-mouse IgG diluted in DPBS containing 0.05% (v/v) Tween 20 and 0.01% (w/v) BSA for 1 h. Embryos were then washed, labeled with PI, mounted on slides and examined as described above.

### Image Analysis using ImageJ

Immunofluorescent intensity was quantified using ImageJ software (version 1.60_41, NIH, Washington DC, USA). For two color images (green for 5-methylcytosine and red for nuclei), individual nuclei were identified by PI labeling and outlined using the free-hand tool on imageJ. Mean gray intensity of the green and red images were determined separately. The ratio of intensity for green and red (5-methylcytosine/DNA) was calculated for each nucleus. Values for all analyzed nuclei from a single embryo were averaged to obtain the average degree of methylation for that embryo. A similar process was used for three-color images except that staining for CDX2 was used to distinguish between TE (blue nuclei) and ICM (no blue labeling) and values were averaged separately for TE and ICM.

### Separation of TE and ICM by Magnetic-activated Cell Sorting

Magnetic-activated cell sorting was performed as previously described with modifications [22]. Blastocysts at d 7 were harvested and incubated in acidic Tyrode’s solution (Millipore, Billerica, MA, USA) to remove zona pellucidae. Zona pellucida free blastocysts were washed three times in MACS buffer [DPBS with 0.5% (w/v) BSA and 2 mM ethylenediaminetetraacetic acid (EDTA), pH 7.2]. Blastocysts were then incubated for 10 min in concanavalin A conjugated with FITC (Sigma-Aldrich, St. Louis, MO, USA; ConA-FITC, 1 mg/ml in MACS buffer). Following three washes in MACS buffer, blastocysts were incubated with 1 µg/ml Hoechst 33342 (Sigma-Aldrich, St. Louis, MO, USA) in MACS buffer for 3 min. Hoechst 33342 labeled blastocysts were then washed three times in MACS buffer and incubated in DPBS containing 1 mM EDTA for 5 min followed by incubation in 0.05% (w/v) trypsin-0.53 mM EDTA solution (Invitrogen Carlsbad, CA, USA) for 10 min at 38.5°C. Groups of 20–30 blastocysts were then disaggregated into single blastomeres by vortexing three times for 10 sec each. Disaggregated blastomeres were transferred into 500 µl DPBS containing 1 mM EDTA and 10% (v/v) fetal bovine serum (Atlanta Biologicals, Norcross, GA, USA) followed by three washes in MACS buffer by centrifugation at 500×g for 5 min. Large blastomere clusters were eliminated by passing the solution through a 0.2 µm cell strainer (BD Biosciences, San Jose, CA, USA). Single blastomeres that passed through the strainer were collected, centrifuged at 500×g for 5 min and resuspended in 110 µl of MACS buffer. The re-suspended solution was incubated with 10 µl magnetic microbeads conjugated to mouse anti-FITC (Miltenyi Biotec, Auburn, CA, USA) for 15 min on ice. Following three washes by centrifugation at 500×g for 5 min, the magnetic bead solution was resuspended in 500 µl MACS buffer and passed through MACS separation columns (Miltenyi biotec, Auburn, CA, USA) attached to a magnetic board (Spherotech, Lake Forest, IL, USA). The FITC-negative fraction (ICM) was eluted by three 500 µl MACS buffer washes followed by FITC positive (TE) elution by removing the MACS separation column from the magnetic board and washing three times with 500 µl MACS buffer. Purity of eluted ICM and TE using this technique is >91% [22].

### Reverse Transcription and Quantitative PCR

Analysis of gene expression was accomplished by quantitative reverse transcription PCR. Pools of embryos were treated with 0.1% (w/v) proteinase from *Streptomyces griseus* to remove the zona pelluicida, washed three times in 50 µl droplets of DPBS with 1% (w/v) PVP, placed in 100 µl extraction buffer from the PicoPure RNA isolation kit (Applied Biosystems, Carlsbad, CA, USA) and heated at 42°C for 30 min. Total RNA was extracted using the PicoPure RNA isolation kit (Applied Biosystems), treated with 2 U of DNase (New England Biolabs, Ipswich, MA, USA) at 37°C for 30 min to remove DNA, and then incubated at 75°C for 15 min to denature DNase. DNase-treated RNA was then reverse-transcribed using the High Capacity cDNA Reverse Transcription Kit (Applied Biosystems). Reverse transcription occurred using random hexamer primers and involved incubation at 25°C for 10 min, 37°C for 120 min and 85°C for 5 min. The cDNA was stored at −20°C until further use. Negative controls for real-time PCR were also performed by incubation without reverse transcriptase.

cDNA was utilized for real-time PCR analysis. For detection of transcript levels, a CFX96 Real-Time PCR detection System (Bio-Rad, Hercules, CA, USA) utilizing SsoFast EvaGreen Supermix with Low ROX (Bio-Rad). Each reaction contained 1 µl forward primer (0.5 µM), 1 µl reverse primer (0.5 µM), 10 µl Evagreen Supermix (Bio-Rad), 6.8 µl H_2_O and 1.2 µl of cDNA sample (0.6 embryo equivalents). Amplification conditions were: 95°C for 30 sec, 40 cycles at 95°C for 5 sec, 60°C for 5 sec, and 1 cycle of melt curve analysis at 65–95°C in increments of 0.5°C every 2 sec. The ΔC_T_ value was determined by subtracting the C_T_ value of the sample by the geometric mean of the C_T_ for three housekeeping genes, *SDHA*, *GAPDH* and *YWHAZ* [23], [24]. The primer sequence for *DNMT3B* (Genbank accession number: AY244711) was 5′-GGGAAGGAGTTTGGAATAGGAG-3′ and 5′-CGGAGAACTTGCCATCACC-3′ [25].

### High Resolution Melting Analysis

The MS-HRM (methylation specific high resolution melting analysis) procedure was based on Wojdacz and Dobrovic [26]. MS-HRM is a procedure that uses an intercalcating dye and sensitive thermocycler to detect single nucleotide changes within an amplicon based on the temperature at which that dsDNA sequence denatures [26], [27]. In the case of methylation analysis, nucleotide changes are induced by bisulfite conversion of cytosines (but not methylcytosine) to uracil and, following DNA replication, thymidine.

As a preliminary step, 0% and 100% methylated standards were generated for use in standard curve generation. For the 0% methylation control, bovine embryonic fibroblast cells derived from skin cells of a fetus at 2–3 mo of gestation were cultured for 1 wk in a culture medium composed of 89% (v/v) Dulbecco’s Modified Eagle’s medium (Invitrogen), 10% (v/v) fetal bovine serum (Atlanta Biologicals, Norcross, GA, USA), 1% (v/v) of a 100 IU/mL penicillin, 100 µg/ml streptomycin sulfate and 250 ng/ml amphotericin B solution (Invitrogen) and 12 µM 5-azacytidine (to inhibit DNA methylation). The concentration of 5-azacytidine used was chosen because it was the highest concentration that did not cause complete loss of cell growth. Other cells were cultured without 5-azacytidine. Cells were collected, stored at −80°C until extraction of DNA and RNA extraction using the AllPrep DNA/RNA Mini kit (Qiagen, Valencia, CA, USA). gDNA and total RNA were stored at −80°C in extraction buffer and RNase-free water, respectively, until ready for use. A sample of 100% methylated DNA was prepared by treating gDNA from fibroblasts cultured in the absence of 12.5 µM 5-azacytidine with SssI methylase (New England Biolabs, Ipswich, MA, USA) for 1 h at 37°C. gDNA was then treated with bisulfite using the Imprint DNA modification kit (Sigma-Aldrich, St. Louis, MO, USA) and frozen at −20°C until further use. Samples of 0 and 100% methylated gDNA were mixed together to create samples of 0, 5, 25, 50, 75 and 100%, methylated gDNA.

High resolution melting analysis was performed using a BioRad C1000 thermal cycler with CFX96 Real-time system and Precision Melt analysis software (BioRad, Hercules, CA, USA). PCR conditions were set to the following: 95°C for 2 min, 45 cycles of 95°C for 10 sec, 60°C for 30 sec, 72°C for 30 sec, and heteroduplex formation at 95°C for 30 sec and 60°C for 1 min. Subsequently, high resolution melting was performed at 0.2°C increments from 65°C to 95°C. Primers were designed using Methyl Primer Express 1.0 (Applied Biosystems, Carlsbad, CA, USA) with settings to include at least one nucleotide toward the 5′ end that could bind to both bisulfite-converted and non-bisulfite converted sequences to decrease bias [28]. Primers were designed to amplify an intronic 81 bp sequence containing 5 CpG. This region of *DNMT3B* was chosen based on the estimation of a CpG island as determined by Methyl Primer Express (Applied Biosystems, Carlsbad, CA, USA). The primer sequence for HRM *DNMT3B* (Genbank accession number NC_007311) were as follows: 5′-GGAYGGGTTTTAGGTTTGGGTATT-3′ and 5′-CAAACCCCCRCAAAATAATTCT-3′. Primers were generated by Integrated DNA Technologies (Coralville, Iowa, USA).

A standard curve was run for each PCR procedure using gDNA of 0–100% methylation and prepared as described above. The difference relative fluorescence units (DRFU) were recorded at the melting peak for each sample. All samples were analyzed in duplicate. The relationship between methylation percent and DRFU was exponential. The standard curve was calculated and used to obtain the degree of methylation for samples.

### Design of Experiments

The first experiment was conducted to determine developmental changes in DNA methylation. Embryos were fertilized with X-sorted spermatozoa (Genex Cooperative, Shawano, WI, USA, Accelerated Genetics, Baraboo, WI, USA and Select Sires, Plain City, OH, USA) from three separate bulls and then placed into culture with SOF-BE1. Separate drops of embryos were cultured to allow harvest of 2-cell embryos [28–32 h post-insemination (hpi)], 4-cell embryos (52–56 hpi), 6–8 cell embryos (76–80 hpi), 9–25 cell embryos (104 hpi), 32 cell-morula stage embryos (148–152 hpi) and blastocyst-stage embryos (176 hpi). Embryos were analyzed for DNA methylation by labeling with anti-5-methylcytosine and PI as described above. The experiment was replicated on 6 occasions and a total of 11–24 embryos per stage were subjected to labeling for anti-5-methylcytosine.

The effect of gender on DNA methylation was determined by fertilizing oocytes with either X-sorted spermatozoa or Y-sorted spermatozoa (Accelerated Genetics, Baraboo, WI, USA) from the same bull, using three separate bulls for each replicate. Embryos were then cultured and harvested from separate drops at either the 6–8 cell stage (76–80 hpi) or blastocyst stage (176 hpi) for analysis of labeling with anti-5-methylcytosine. The experiment was replicated on 3 occasions and a total of 11–21 embryos per stage were subjected to labeling for anti-5-methylcytosine.

Effects of CSF2 and cell lineage (ICM and TE) on DNA methylation in the blastocyst were tested as follows. Oocytes were fertilized with either X-sorted spermatozoa or Y-sorted spermatozoa (Accelerated Genetics, Baraboo, WI, USA) from the same bull, using three separate bulls for each replicate. Embryos were then placed in 45 µl drops of SOF-BE1 and cultured for 7 d. At 5 d pi, 5 µl of 100 ng/ml recombinant bovine CSF2 (Novartis) in vehicle [DPBS and 1% (w/v) BSA] or vehicle alone was added. The concentration of CSF2 was chosen based on its effectiveness for increasing development to the blastocyst stage and embryonic survival after transfer into recipient females [15], [29]. Blastocysts were harvested at Day 7 pi and subjected to labeling with anti-5-methylcytosine, anti-CDX2 and PI. The experiment was replicated on 3 occasions and a total of 14–26 embryos per stage were subjected to labeling for anti-5-methylcytosine.

Developmental changes in expression of *DNMT3B* were determined in another experiment. Embryos were fertilized with conventional semen (Androgenics, Oakdale, CA, USA and Nebraska Bull Service, McCook, NE, USA) using three different bulls for each replicate and then placed into culture with SOF-BE1. Separate drops of embryos were cultured to allow collection of groups of 30 zygotes (0 hpi), 2-cell (28–32 hpi), 3–4 cell (48 hpi), 5–8 cell (56–60 hpi), 9–16 cell (72 hpi), >16 cell (120 hpi) and blastocyst stage (168 hpi) embryos. RNA was extracted as described above and used for RT-PCR. The experiment was replicated on 5 occasions.

Methylation of an intronic region of *DNMT3B* was determined in two experiments. In the first experiment, embryos were collected at the 6–8 cell stage (76–80 hpi) and at the blastocyst stage (176 hpi). In the second, blastocysts were harvested at 176 hpi and ICM and TE isolated using MACS. In both experiments, embryos were fertilized with conventional semen (Androgenics, Oakdale, CA, USA and Nebraska Bull Service, McCook, NE, USA) using three different bulls for each replicate and then placed into culture with SOF-BE1. In the first experiment, embryos were collected in groups of 18–48, treated to remove the zona pellucida and snap-frozen in liquid N_2_ before storage at −80°C until ready for use in MS-HRM analysis. A total of 3 pools of embryos were collected at each stage of development. In the second experiment, groups of 20–40 blastocysts were subjected to the MACS procedure to isolate ICM and TE and cells frozen in 20 µl of MACS buffer (DPBS with 0.5% (w/v) BSA and 2 mM EDTA, pH 7.2) at −80°C until ready for MS-HRM. The experiment was replicated with 8 pools of ICM and TE.

### Statistical Analysis

Data were analyzed by least-squares analysis of variance using the GLM procedure of the Statistical Analysis System version 9.3 (SAS Institute Inc., Cary, NC, USA). Depending on the experiment, sources of variation included replicate, stage, gender, cell type (ICM and TE), treatment (CSF2 or vehicle) as well as all interactions. Replicate was considered a fixed effect. For real-time PCR results, treatment effects were tested using ΔC_T_ values and graphed as fold-change differences relative to values for oocytes. All data are shown as least-squares means ± standard error of the means.

## Results

### Changes in DNA Methylation during Development to the Blastocyst Stage

**Figure 1:**
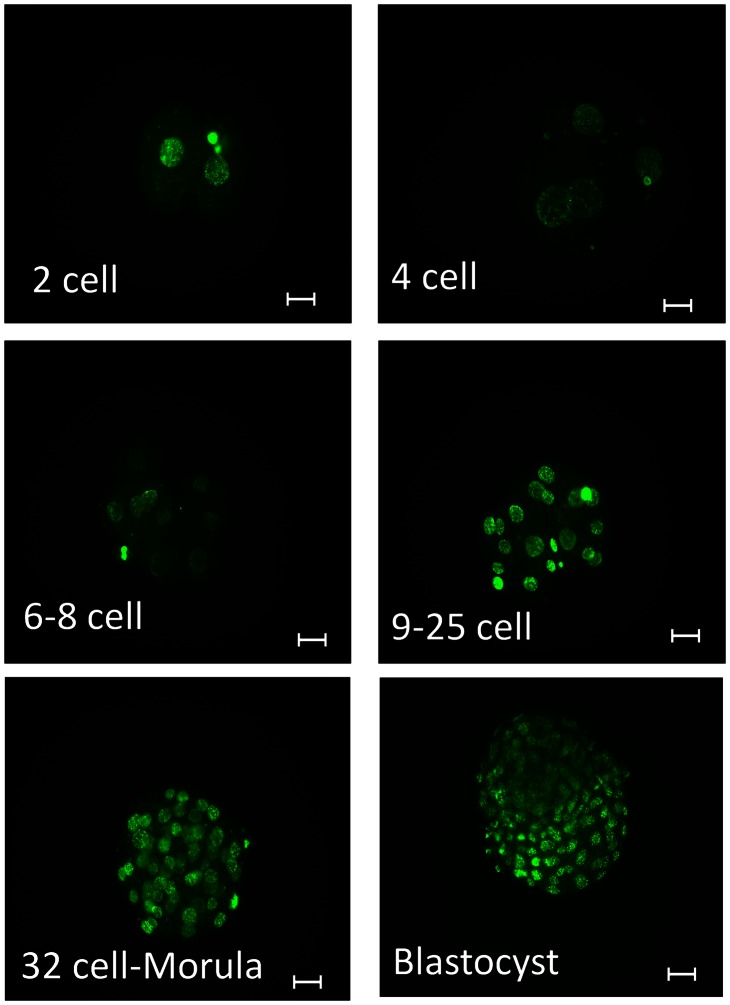
Representative images of embryos labeled for immunoreactive 5- methylcytosine at the 2 cell, 4 cell, 6–8 cell, 9–25 cell, 32 cell-morula and blastocyst stages of development. The scale bar represents 26.75 µm.

**Figure 2:**
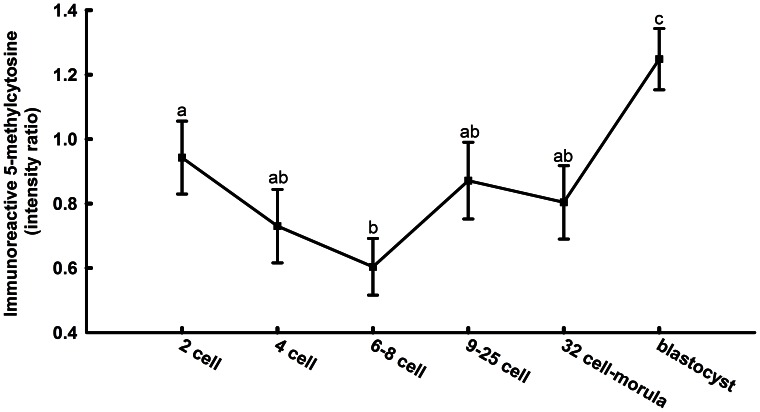
Developmental changes in labeling of immunoreactive 5-methylcytosine from the 2-cell to blastocyst stages of development. Data represent the ratio of fluorescent intensity for anti-5-methylcytosine to that for propidium iodide. There was an overall effect of stage on labeling intensity (P<0.0001). Means with different superscripts differ (P<0.05). Data are least-squares means±SEM of results from 11–24 embryos per stage.

Representative images of labeling for 5-methylcytosine from the 2-cell stage through blastocyst development are presented in Figure 1 while results from quantitative analysis are presented in Figure 2. There was an effect of stage of development on immunoreactive 5-methylcytosine (P<0.0001), with amounts decreasing from a peak at the 2-cell stage to a nadir at the 6–8 cell stage. Thereafter, immunoreactivity increased to the blastocyst stage.

### Effect of Gender on DNA Methylation

**Figure 3:**
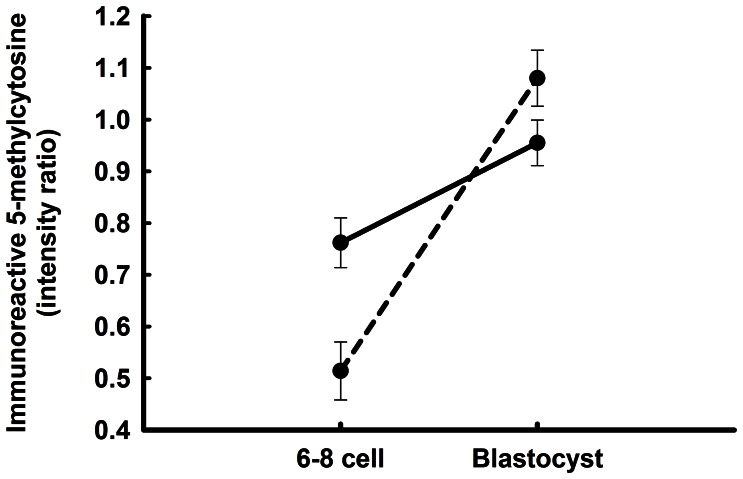
Effects of gender on immunoreactive 5-methylcytosine in embryos at the 6–8 cell and blastocyst stages of development. Data represent the ratio of fluorescent intensity for anti-5-methylcytosine to that for propidium iodide. The solid line represents female embryos while the hashed line represents male embryos. Data are least-squares means±SEM of results from 11–21 embryos per stage. Amounts of DNA methylation were affected by gender (P = 0.0029), stage of development (P<0.0001) and the stage × gender interaction (P = 0.0007).

Embryos were fertilized separately with X and Y sorted sperm from the same bull to produce either female or male embryos. DNA methylation was examined at either the 6–8 cell or blastocyst stages of development by evaluating labeling with anti-5-methylcytosine. Results are shown in Figure 3. DNA methylation was greater for embryos at the blastocyst stage than at the 6–8 cell stage (stage of development; P<0.0001). There were, however, effects of gender (P = 0.0029) and gender × stage (P = 0.0007). The interaction occurred because female embryos had more intense labeling for 5-methylcytosine than male embryos at the 6–8 cell stage but lower labeling than male embryos at the blastocyst stage.

### DNA Methylation in Blastocysts as Modulated by Gender, CSF2 and Cell Differentiation

**Figure 4:**
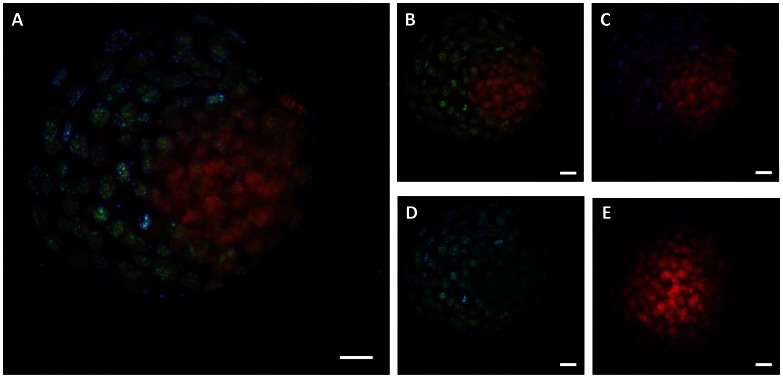
Representative images of an embryo in which labeling with CDX2 (blue) was used to evaluate immunoreactive 5-methylcytosine (green) in ICM (CDX2-negative) and TE (CDX2-positive). Nuclei of all cells were labeled with propidium iodide (red). Panels represent merged images for all three fluorescent labels (A), 5-methylcytosine and PI (B), CDX2 and PI (C), CDX2 and 5-methylcytosine (D) and control antibodies for anti-5methylcytosine and anti-CDX2 as well as PI (E). The scale bar is 20 µm.

**Figure 5:**
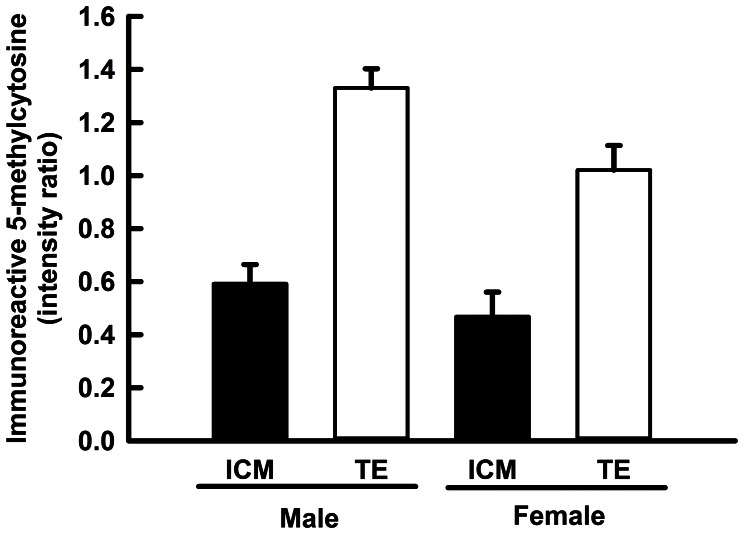
Effects of gender and cell type [inner cell mass (ICM) and trophectoderm (TE)] on immunoreactive 5-methylcytosine in blastocysts. Data represent the ratio of fluorescent intensity for anti-5-methylcytosine to that for propidium iodide. Data are least-squares means±SEM of results from 14–26 embryos per gender. Amounts of DNA methylation were affected by gender (P<0.02), and cell type (P<0.0001) but not the gender × cell type interaction (P<0.25).

Use of CDX2 to distinguish between ICM and TE revealed that fluorescent activity for immunoreactive 5-methylcytosine was lower in ICM than TE. A representative blastocyst is shown in Figure 4 and quantitative analysis is summarized in Figure 5. As seen in the previous experiment, DNA was less methylated in female embryos than male embryos (P<0.02) and this was apparent in both the ICM and TE compared to the TE (Figure 5). There was no effect of CSF2 or interactions of CSF2 on degree of methylation in either the ICM (0.53±0.09 vs. 0.46±0.07 for CSF2 and vehicle, respectively) or TE (1.17±0.09 vs. 1.18±0.07 for CSF2 and vehicle, respectively).

### Developmental Changes in *DNMT3B* Gene Expression

**Figure 6:**
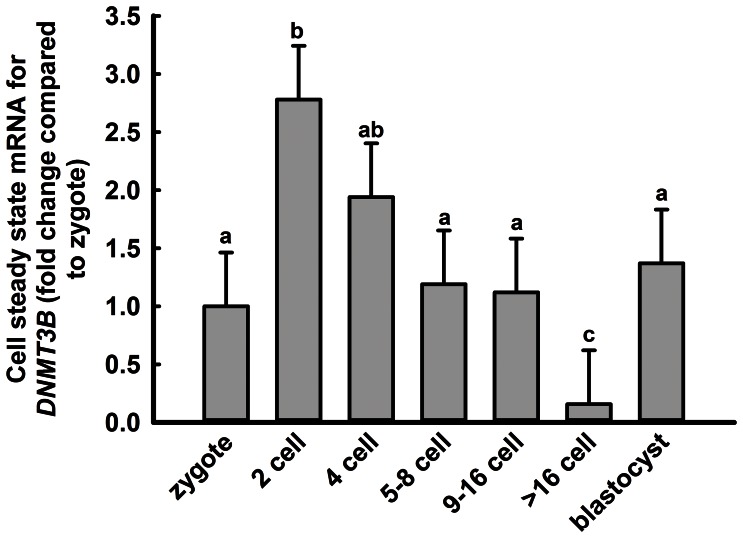
Developmental changes in steady-state mRNA for *DNMT3B*. Data for mRNA are expressed as fold-change relative to amounts for zygotes and represent the least-squares means±SEM of results from 5 pools of 30 embryos per stage. Means with different superscripts differ (P<0.05).

To determine whether developmental changes in DNA methylation could conceivably be caused by changes in DNMT3B activity, changes in expression of *DNMT3B* from the zygote to the blastocyst stage were determined (Figure 6). Stage of development affected (P<0.0001) steady-stage amounts of DNMT3B mRNA. Expression increased between the zygote stage and 2-cell stage and then declined to a nadir for embryos at the >16 cell stage of development. Thereafter, steady-state concentrations of *DNMT3B* increased at the blastocyst stage.

### Developmental Changes in Methylation of *DNMT3B*

**Figure 7:**
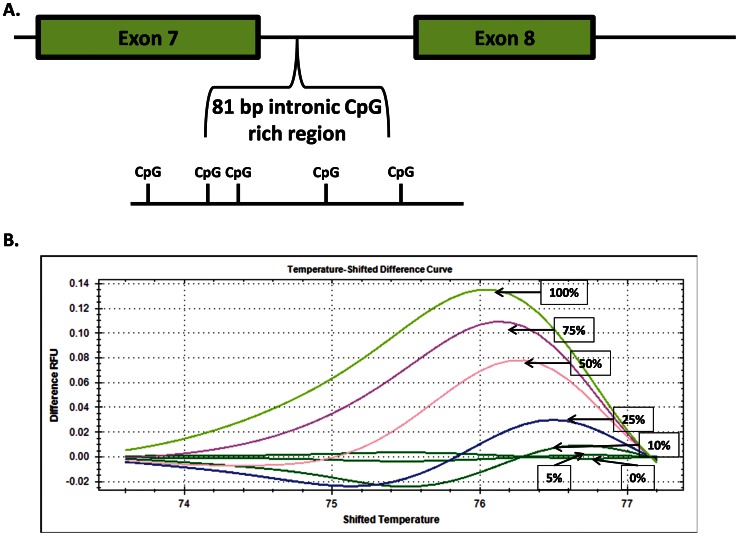
Characteristics of the high resolution melt analysis for methylation of an intronic region of *DNMT3B*. Panel A shows the location of the intronic region between exons 7 and 8 that was analyzed. Note the presence of five CpG eligible for methylation in the 81 bp region amplified by PCR. Panel B is a representative result for the standard curve generated by analysis of a mix of control DNA at 0% and 100% methylation. The graph is a plot of the difference relative fluorescence units (RFU) as a function of shifted temperature (X-axis normalization to reduce variation between wells).

**Figure 8:**
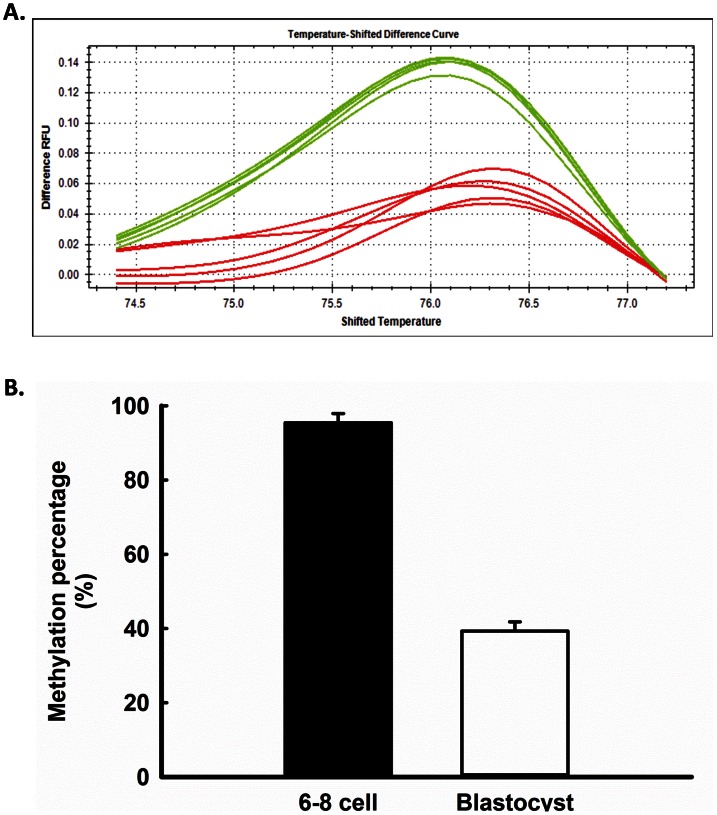
Developmental changes in DNA methylation for *DNMT3B*. Panel A is a representative result of analysis of samples from 6–8 cell embryos (lime green) and blastocysts (red). Panel B shows the degree of methylation between the two stages. Data represent least-squares means±SEM of results from 3 pools of 23–48 embryos per stage. Methylation percent was higher at the 6–8 cell stage than at the blastocyst stage (P<0.0001).

The possible role of methylation in controlling developmental changes in expression of *DNMT3B* was evaluated by determining changes in the degree of methylation in an 81 bp CpG rich intronic region of *DNMT3B* (Figure 7A). A representative standard curve is shown in Figure 7B and representative results in Figure 8A. Methylation in the analyzed region, which contains 5 CpGs, was higher (P<0.0001) for 6–8 cell embryos than for embryos at the blastocyst stage (Figure 8B). When ICM and TE were separated by MACS, there was no significant difference (p = 0.25) in methylation of *DNMT3B* between ICM (70.2±9.8%) or TE (56.3±8.6%).

## Discussion

Epigenetic control of gene expression is an important aspect of early embryonic development. Using the preimplantation bovine embryo as a model, it was observed that dynamic changes in DNA methylation occur throughout development in a manner that depends on gender and cell type. Changes in DNA methylation were associated with similar changes in expression and methylation of *DNMT3B,* indicating that, as for the mouse [5], this gene may play an important role in modification of the embryonic methylome.

There is a steady decrease in overall level of DNA methylation as the mouse embryo undergoes successive cell division [30]. A similar loss occurs in the developing sheep embryo [8] and as, shown here, the bovine embryo (Figure 2). In contrast, there appears to be no widespread demethylation during early cleavage divisions in the pig [9] or rabbit [10]. In the mouse, the loss of methylation during early development occurs as a result of both passive [31] and active [32] demethylation. Passive demethylation occurs when DNA replication occurs without the presence of DNMT1 to methylate the newly replicated strand [33] while active DNA demethylation is thought to occur by base excision repair mechanisms utilizing glycolysis or deamination of 5-methylcytosine although the exact enzymes responsible are still unknown in mammals [34].

The bovine embryo is also similar to the mouse embryo in that the period of demethylation is followed by a period of *de novo* methylation (Figure 2). In the cow, methylation begins following the 6–8 cell stage and is coincident with activation of the embryonic genome [35]. Re-establishment of methylation marks on DNA in the mouse embryo is accomplished by the *de novo* methyltransferases Dnmt3a and Dnmt3b [5]. Knockout of *Dnmt3b* caused incomplete methylation of pluripotent genes in the mouse embryo [5], [36]. Current results implicate DNMT3B as involved in *de novo* DNA methylation in the cow based on the observation that steady-state amounts of mRNA for *DNMT3B* parallels the pattern of methylation (Figures 2 and 6). In fact, *DNMT3B* itself might be under the control of methylation because methylation of an intronic region within *DNMT3B* was lower at the blastocyst stage than at the 6–8 cell stage (Figure 8B). The role of methylation in *DNMT3B* expression can be further clarified through experiments to detail the methylation landscape of *DNMT3B* more fully as well as the consequences of changes in methylation on *DNMT3B* expression. It is also likely that there is regulation of *DNMT3B* transcription independent of DNA methylation.

Developmental patterns of methylation in the preimplantation embryo diverge between the mouse and cow at the blastocyst stage of development. In the mouse, the ICM is more highly methylated than the TE [4], [37] while current results indicate the ICM is less methylated than TE in the cow (Figure 4). Indeed, there is much divergence between species in overall levels of methylation in the ICM and TE, with the sheep and pig showing a pattern similar to the mouse [8], [9] and with the ICM less methylated than the TE in the rabbit [10], [38]. One implication of the species variability in DNA methylation patterns in the blastocyst is that there is likely to be significant differences between species in the genes that are differentially regulated between ICM and TE. One example is *OCT4* (i.e, *POU5F1*). In the mouse, *Oct4* is strictly expressed in the ICM while *OCT4* in cattle is expressed in both ICM and TE [39]. A genome-wide study of the bovine ICM and TE revealed genes that exhibited different patterns of differential expression than is the case in mice or humans [40].

Gender of the bovine embryo is an important factor that affects development as early as the first cleavage, when the sex ratio is skewed towards males [41]. *SRY* is expressed as early as the 8-cell stage of development [42], more male embryos than female embryos develop past the 8-cell stage of development [43], and male embryos become blastocysts at a more rapid rate than females [41], [43], [44]. At the blastocyst stage, gene expression is affected by gender, with the overall level of transcription being greater for female embryos [13]. Methylation is likely to play a role in establishing gender patterns of transcription. Earlier studies reported differences in degree of methylation of specific genes at the blastocyst stage [14], [45], [46]. Here we show that the overall level of DNA methylation at the blastocyst stage was lower for female embryos than male blastocysts (Figure 3). Such a gender difference would be expected if DNA methylation is important for the higher transcript abundance for female blastocysts [13]. Earlier in development, at the 6–8 cell stage, DNA is more methylated for female embryos (Figure 3), probably because the rate of demethylation was less for female embryos.

DNA methylation can be altered by development in vitro [46]. Thus, it will be necessary to determine the extent to which changes in DNA methylation caused by stage of development, cell lineage and gender seen here also occur for embryos developing in vivo. Moreover, it is possible that regulatory molecules such as CSF2 [15], insulin-like growth factor-1 [16] and hyaluronan [17] that improve competence of the in vitro produced embryo to survive after transfer into recipients could exert their action, at least in part, by regulating DNA methylation. This hypothesis was tested in the present experiment for the maternally derived cytokine CSF2 [47]. There was no effect of CSF2 treatment on the overall level of DNA methylation at the blastocyst stage (Figure 3C) so actions of CSF2 on the embryo that enhance its survival probably do not depend upon broad changes in DNA methylation. Nonetheless, it is possible that CSF2 causes changes in methylation of a more narrow set of genes. There are long-term consequences of embryonic treatment with CSF2 (decreased pregnancy loss after Day 35 of gestation [15] that could represent epigenetic programming.

In conclusion, preimplantation development of the bovine embryo is characterized by dynamic changes to DNA methylation that are dependent upon gender and cell lineage. In particular, global methylation declines to a nadir at the 6–8 cell stage and increases thereafter. Methylation is lower for female embryos than male embryos at the blastocyst stage and lower for the ICM than TE. The developmental pattern of DNA methylation in the cow is partially representative of events in the mouse, with the major difference being in the relative degree of methylation in ICM and TE. Like the mouse, changes in expression of *DNMT3B* may be responsible for developmental changes in DNA methylation because levels of methylation are related to expression of *DNMT3B.*
